# Structural Evolution and Mechanical Properties of CrSiN/WSiN Multilayer Coatings

**DOI:** 10.3390/ma19183837

**Published:** 2026-09-09

**Authors:** Li-Chun Chang, Yan-Zhi Liao, Yu-Ting Ye, Yung-I Chen

**Affiliations:** 1 Department of Materials Engineering, Ming Chi University of Technology, New Taipei 243303, Taiwan; lcchang@mail.mcut.edu.tw; 2 Center for Plasma and Thin Film Technologies, Ming Chi University of Technology, New Taipei 243303, Taiwan; 3 Department of Optoelectronics and Materials Technology, National Taiwan Ocean University, Keelung 202301, Taiwan; 11289034@mail.ntou.edu.tw (Y.-Z.L.); yuting900815@gmail.com (Y.-T.Y.)

**Keywords:** mechanical properties, multilayer coatings, wear resistance

## Abstract

Monolithic CrSiN and WSiN films, as well as multilayered CrSiN/WSiN coatings, were fabricated via magnetron sputtering. The effects of bilayer stacking periods on the multilayer coatings’ characteristics were investigated. The monolithic CrSiN and WSiN films exhibited crystalline and near-amorphous structures, respectively, whereas the multilayer coatings exhibited mixed crystalline and amorphous phases. Moreover, the crystallinity of individual sublayers was affected by the underlying sublayers. The multilayer architecture effectively combined the mechanical and tribological properties of constituent sublayers. The mechanical properties of the multilayer coatings increased with decreasing bilayer period. The multilayered CrSiN/WSiN coatings with 12 alternative sublayers and a bilayer period of 143 nm exhibited a hardness and elastic modulus of 20.7 and 257 GPa, respectively. The bilayer and four-layer coatings demonstrated excellent wear resistance. Overall, the design of CrSiN/WSiN multilayer coatings with an optimized bilayer period can significantly enhance mechanical strength and wear resistance, indicating promising potential for advanced protective coating applications.

## 1. Introduction

A material’s structure affects its characteristics. Thin films without a dendritic structure, formally seen in a cast bulk form, exhibit high mechanical properties and are widely applied for surface modification [1]. Transition-metal nitride (TMN) and Si-containing TMN coatings have been developed over recent decades to protect the underlying materials against mechanical [2,3,4,5,6], oxidative [3,5], and corrosive [2,5] impacts in severe environments. The Si-containing TMN coatings with nanocomposite structures have attracted significant attention due to the coexistence of immiscible nanocrystalline and amorphous phases [2,3,4,5,6]. The incorporation of Si into TMN results in solid-solution strengthening, grain refinement of the nitride grains, and amorphous SiN*_x_* phase formation, thereby suppressing deformation through dislocation motion [6,7,8,9,10]; therefore, it effectively improves the mechanical and antioxidative properties of the TMN films. However, when excessive Si content was added, the volume of the amorphous phase increased notably, thereby degrading the mechanical properties [11]. Moreover, alterations in thin-film technology affect the film’s mechanical properties. CrSiN films with hardness values of 22 and 31.5 GPa have been reported for films fabricated by conventional sputtering [12] and high-power impulse magnetron sputtering [6], respectively. In a previous study [13], the nanocomposite Cr_32_Si_14_N_54_ film, prepared without external heating or bias voltage, had a hardness of 17 GPa. Moreover, the Cr_34_Si_14_N_52_ film possessed a higher hardness of 22.2 GPa under the assistance of −100 V substrate bias and 400 °C substrate temperature [14]. In contrast, the amorphous WSi_1–14_N films had higher hardnesses ranging from 19.5 to 24.5 GPa [15].

Multilayer coatings exhibit mechanical and tribological properties superior to conventional monolayer films [16,17,18,19,20,21,22], making them the focus of extensive scientific research and industrial applications. The multilayer architecture enhances hardness and toughness through various strengthening mechanisms, comprising dislocation blocking at interlayer interfaces, Hall–Petch-like effect due to layer refinement, and crack deflection at interfaces [23,24]. The dense network of interfaces in multilayers alters crack propagation paths, significantly improving the coatings’ fracture resistance and wear durability [25,26,27]. Studies have shown that alternating layers with varying composition, thickness, and interfacial characteristics lead to pronounced hardness enhancement as the bilayer period decreases, primarily due to the hindrance of dislocation glide by the multiple interfaces [28,29]. Multilayer coatings can be fabricated through magnetron sputtering, vacuum-arc evaporation, arc ion plating, chemical-vapor deposition, and electrodeposition [30,31]. Magnetron sputtering was widely used to fabricate multilayers consisting of transition and refractory metals and their nitrides such as TiN/VN [32], CrN/TiN [18], TiN/W_2_N [20], Cr/CrN [33], MoN/MoSiN [22], TiAlSiN/VSiN [34], and Cr/CrAlN [35] multilayer coatings. In a previous study [36], CrSiN/WSiN bilayer films were fabricated that combined the antioxidant and antiwear advantages of the CrSiN layer with the mechanical properties of the WSiN layer. The 600 °C annealed CrSiN/WSiN bilayer films exhibited nitrogen loss, leading to a phase evolution from CrN to Cr_2_N. Moreover, the formed surface SiO_2_ and Cr-Si-O layers were amorphous and served as protective oxide scales, effectively suppressing further oxidation in bilayer films with a 539 nm CrSiN sublayer and a CrSiN-to-WSiN sublayer thickness ratio of 0.77. The decomposition of WSiN sublayers, resulting in the formation of a W phase, was also observed. Moreover, the outward diffusion of W led to WO_3_ formation in bilayer films with lower CrSiN sublayer thicknesses of ≤250 nm or CrSiN-to-WSiN sublayer thickness ratios of ≤0.32. The porous WO_3_ oxide layers are prone to spallation due to the significant volumetric expansion associated with the transformation from W to WO_3_. Therefore, designing multilayered structures that sustain a high CrSiN-to-WSiN sublayer thickness ratio was expected to reduce W-induced damage during annealing. This study fabricated CrSiN/WSiN multilayer coatings with various bilayer periods for use as protective hard coatings. The enhancements in mechanical and tribological properties were investigated by introducing the multilayer architecture.

## 2. Materials and Methods

CrSiN/WSiN multilayer coatings with a Cr interlayer were fabricated. The targets, Cr, W, and Si, had a diameter of 50.8 mm. The substrates were arranged on an electrically grounded holder. The holder was not externally heated and was rotated at 30 rpm during the sputtering [36]. The samples prepared on SUS304 substrates were used for the scratch and wear tests, whereas all other tests were performed on samples prepared on Si substrates. The SUS304 substrates were ground and polished using a 0.3 μm Al_2_O_3_ slurry. All the substrates were ultrasonically cleaned in alcohol and acetone for 15 min each before being placed in the vacuum chamber. The Cr interlayer was deposited under a working pressure of 0.4 Pa, an Ar flow of 20 sccm, and a direct current (DC) power of 150 W for 10 min. The Cr interlayer improved adhesion between the coating and the substrates [36] and reduced differences in coating quality across substrates. Then, N_2_ and Ar flows of 8 and 12 sccm were regulated to deposit Si-containing nitride sublayers. WSiN and CrSiN sublayers were cosputtered sequentially to fabricate the multilayer coatings. The WSiN sublayers were deposited using DC powers of 150 and 100 W on W and Si targets, respectively. In contrast, the CrSiN sublayers were coated using a DC power of 100 W on both Cr and Si targets. The CrSiN/WSiN multilayer coatings with 2, 4, 8, and 12 alternating sublayers were fabricated and designated as samples 2L, 4L, 8L, and 12L, respectively. The substrate temperature was raised to 60 °C because of energetic particle impaction during sputtering. Table 1 lists the deposition durations for the individual sublayers. The monolithic CrSiN and WSiN films were prepared for comparison, with chemical compositions of 32.2% Cr–11.4% Si–55.7% N–0.7% O and 48.9% W–9.5% Si–41.1% N–0.5% O, respectively, represented as Cr_32_Si_12_N_56_ and W_49_Si_10_N_41_.

The phases of the multilayer coatings were analyzed using XRD (X’Pert PRO MPD, PANalytical, Almelo, The Netherlands) with Cu Kα radiation. The coatings’ lattice constants (*ɑ*_0_) were determined from the grazing incident XRD reflections by using the following equation [37]:

(1) $$a=a_{0}+K\times \frac{\cos^{2}\theta }{\sin\theta }$$

where *ɑ* is the lattice constant for an individual reflection, *K* is a constant, and *θ* is the diffraction angle. Field emission scanning electron microscopy (FE–SEM) was used to determine the layer thicknesses. The nanostructures were examined using a transmission electron microscope (TEM, JEM-2010F, JEOL, Tokyo, Japan). The TEM samples were prepared using a focused ion beam system (NX2000, Hitachi, Tokyo, Japan). The hardness and elastic modulus of the multilayer coatings were measured using a nanoindentation tester (TI–900 Triboindenter, Hysitron, Minneapolis, MN, USA) with a Berkovich diamond probe tip (100 nm radius) and a depth of 80 nm, and calculated using the Oliver–Pharr method [38]. The loading, holding, and unloading times were all 5 s. The Poisson ratios of the coatings were assumed to be 0.25 [36,39,40] for calculating the coatings’ elastic modulus. The curvature method was used to measure the coatings’ residual stress [41]. The adhesion strength between the multilayer coatings and the SUS304 substrate was evaluated using a scratch tester [42]. The wear resistance was assessed using a pin-on-disk test, in which a 6 mm tungsten carbide (WC–6 wt.% Co) ball served as the stationary pin. The hardness of the tungsten carbide ball was 14.2 GPa, comparable to the hardness level of the studied coatings. The normal load, sliding speed, and track diameter were set at 1 N, 104.5 mm/s, and 20 mm, respectively. The wear depth and volume were measured using white-light interferometry (WLI, Profilm3D, Filmetrics, San Diego, CA, USA).

## 3. Results and Discussion

### 3.1. Phase Structure

**Figure 1:**
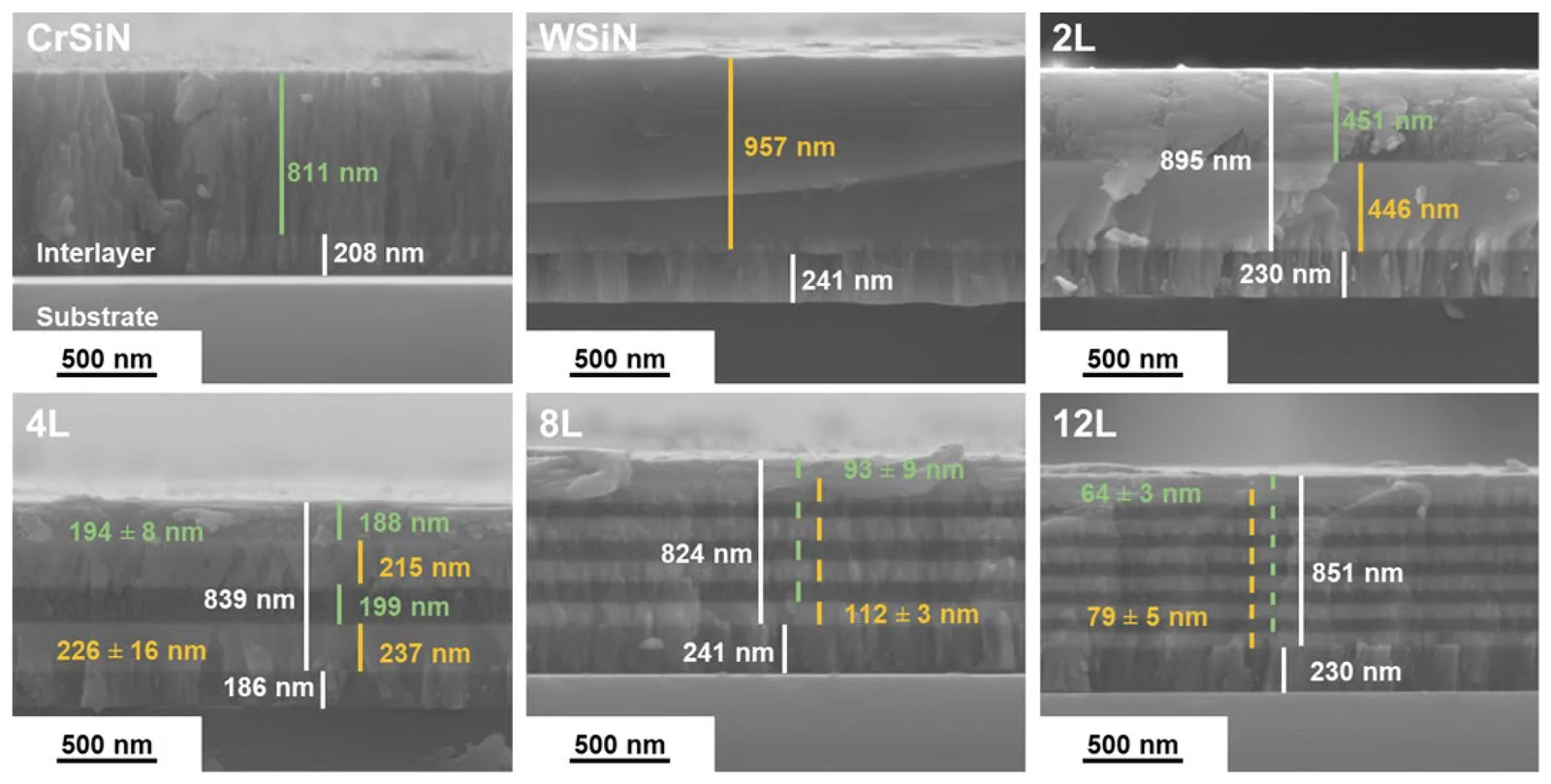
XSEM images of the CrSiN/WSiN coatings exhibit the thicknesses of the individual sublayers.

Figure 1 displays the cross-sectional SEM (XSEM) images of CrSiN/WSiN multilayer coatings. A Cr interlayer of 186–241 nm was introduced beneath the first (WSiN) sublayer to enhance interfacial adhesion to the substrate. The thickness values of each sublayer were regulated by considering the deposition rates of each monolithic layer. The deposition rates were 11.26 and 11.39 nm/min for the monolithic CrSiN and WSiN films, respectively, indicating a thickness ratio T_CrSiN_/T_WSiN_ of 0.99 for these multilayer coatings; for example, sample 2L exhibited a thickness ratio T_CrSiN_/T_WSiN_ of 1.01, close to the predicted value of 0.99. However, this thickness ratio decreased as the number of stacking layers increased. The individual thickness values of the CrSiN and WSiN sublayers are summarized in Table 1. The T_CrSiN_/T_WSiN_ ratios decreased from 1.01 to 0.86, 0.83, and 0.81 as the number of stacked sublayers increased from 2 to 4, 8, and 12, respectively. The bilayer periods of samples 2L, 4L, 8L, and 12L were 897, 420, 205, and 143 nm, respectively. The morphologies of the monolithic CrSiN and WSiN films were crystalline and featureless, respectively.

**Figure 2:**
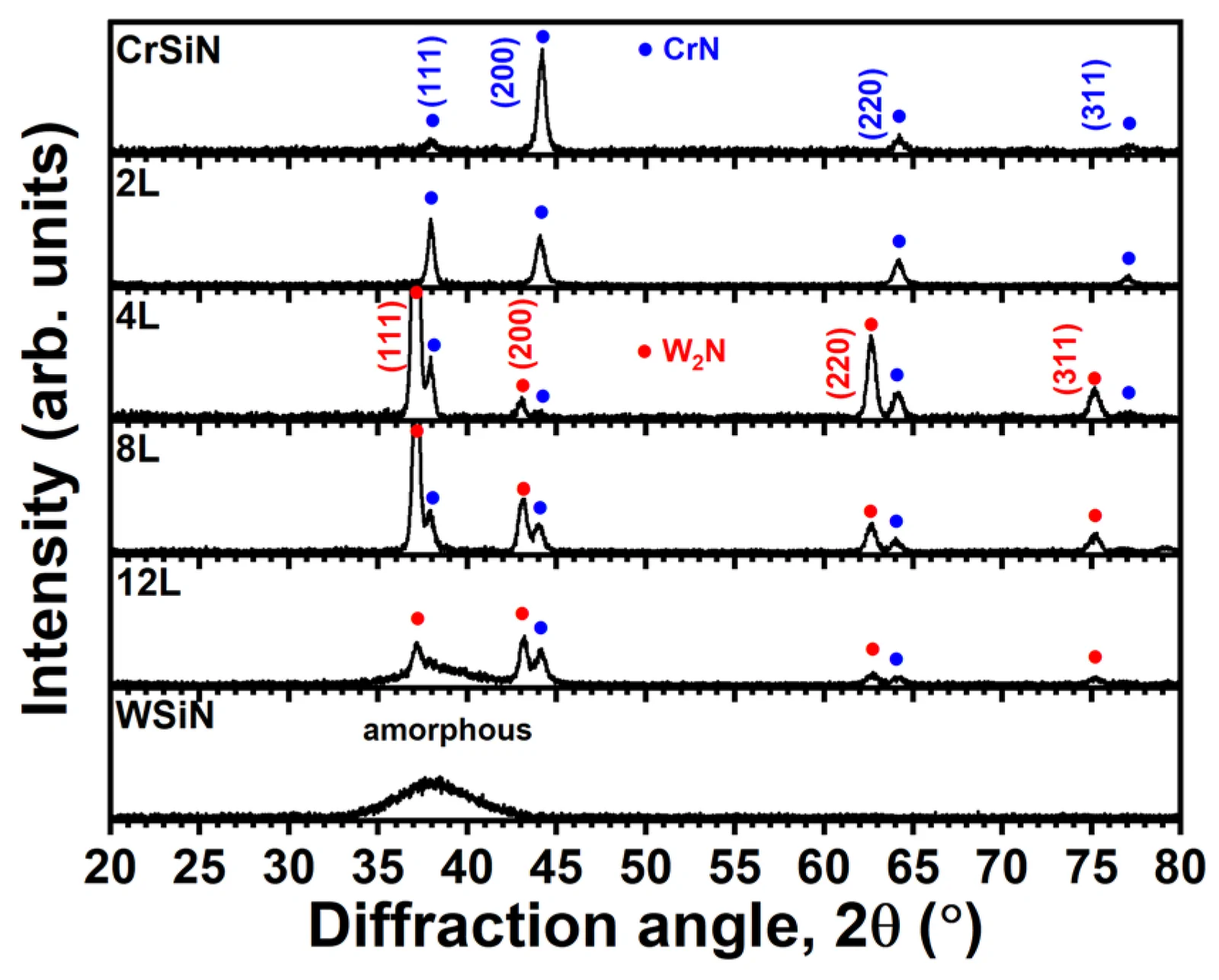
GIXRD patterns exhibit phase structures of the CrSiN/WSiN coatings. CrN, W_2_N, and amorphous phases are indicated.

Figure 2 depicts the GIXRD patterns of the CrSiN/WSiN multilayer coatings and the monolithic CrSiN and WSiN films. The monolithic Cr_32_Si_12_N_56_ film exhibits reflections of a face-centered cubic (FCC) CrN phase with a lattice constant of 0.4102 nm, which was smaller than 0.4140 nm (ICDD 00-011-0065) for the standard CrN phase, implying the substitution of Si for Cr in the lattice, accompanied by a shrinkage of 0.9% in the lattice constant. The reported solubility of Si in the CrN lattice was <4.8 at.% [2,8,12,43], which implied that the additional Si atoms in the nitride film formed an amorphous SiN*_x_* phase. By contrast, the monolithic WSiN film displays an amorphous structure. The 2L sample, a bilayer film, exhibits a mixture of FCC and amorphous phases, which is attributed to the stacking sequence of the Cr interlayer, WSiN sublayer, and CrSiN sublayer. In contrast, samples 4L, 8L, and 12L, the typical multilayer coatings consisting of alternating WSiN and CrSiN sublayers, have an additional cubic W_2_N (ICDD 00-025-1257) phase, which is attributed to the stacking of WSiN sublayers on the underlying crystalline CrSiN sublayers and the similar lattice constants between W_2_N and CrN phases.

**Figure 3:**
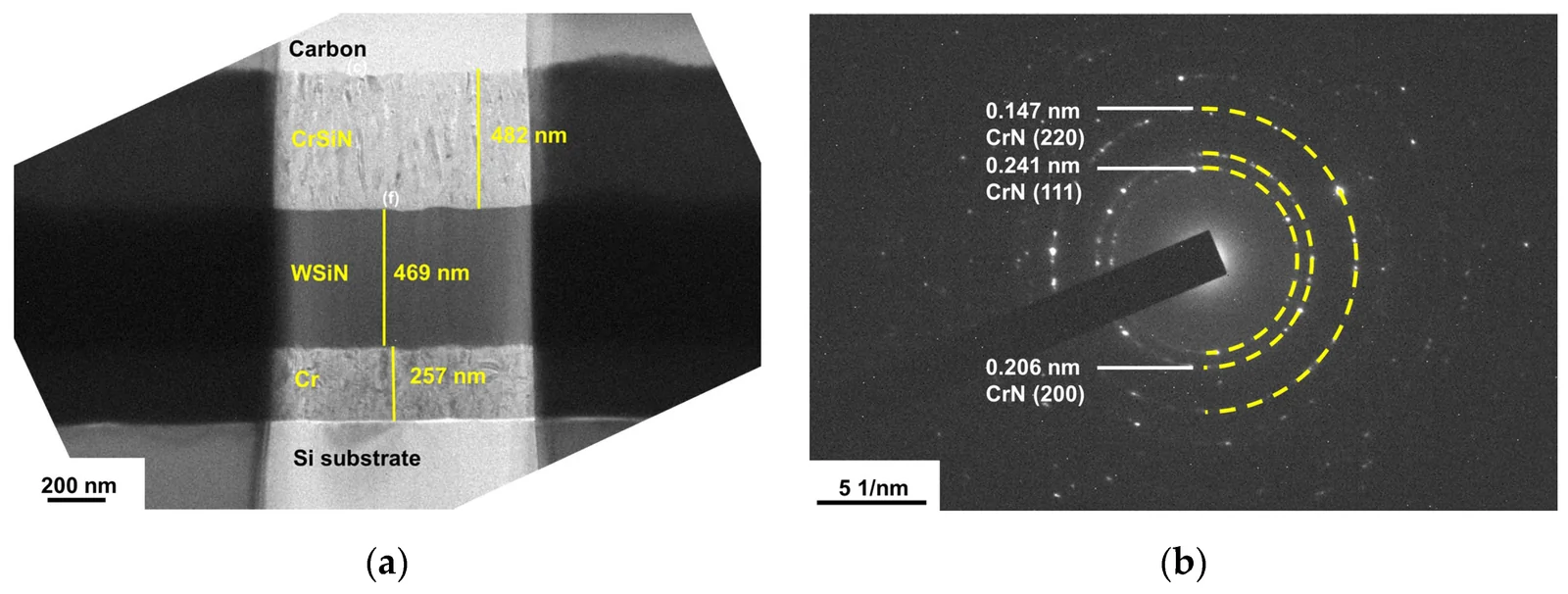

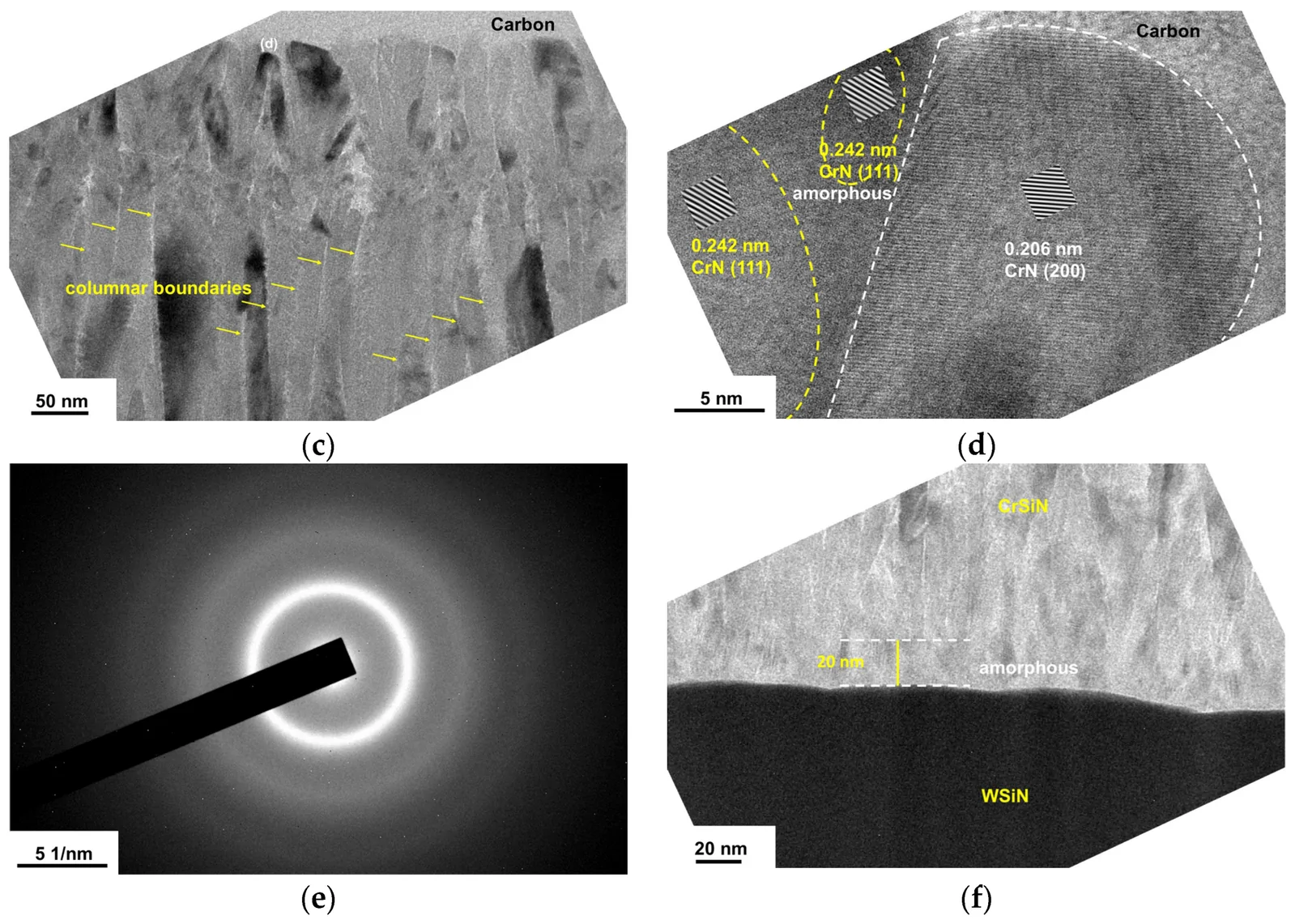
(**a**) The XTEM image displays the stacking of Cr, WSiN, and CrSiN sublayers of sample 2L. (**b**) The SAED pattern of the CrSiN sublayer reveals an FCC crystalline phase. (**c**) The yellow arrows point out the columnar boundaries in the magnified XTEM image of the CrSiN sublayer at the position labeled in (**a**). (**d**) The HRTEM image of the CrSiN sublayer shows crystalline and amorphous regions. (**e**) The SAED pattern indicates the WSiN sublayer’s near-amorphous structure. (**f**) The XTEM image at the CrSiN/WSiN interface exhibits an amorphous structure at the initial growth of the CrSiN sublayer above the WSiN sublayer.

Figure 3a presents the cross-sectional TEM (XTEM) image of sample 2L, stacking with a 257 nm-thick Cr interlayer, a 469 nm-thick WSiN sublayer, and a 482 nm-thick CrSiN sublayer, with layer thicknesses closely matching those shown in the XSEM image (Figure 1). Figure 3b shows a selected area electron diffraction (SAED) pattern of the CrSiN sublayer in sample 2L, where the diffraction rings correspond to the (111), (200), and (220) planes of an FCC CrN phase, with interplanar spacings (*d*-spacings) of 0.241, 0.206, and 0.147 nm, respectively. The magnified TEM image (Figure 3c) showed that the CrSiN sublayer exhibited a columnar structure tens of nanometers wide, indicated by yellow arrows, with clearly defined grain boundaries. The high-resolution TEM (HRTEM) image of the CrSiN layer (Figure 3d) confirms a well-crystallized CrN phase, as evidenced by distinct lattice fringes. Selected zones with dimensions of 2.4 × 2.4 nm exhibit lattice fringes of 0.242 and 0.206 nm for the (111) and (200) planes of the CrN phase, respectively. The dashed-line-enveloped regions indicate crystalline domains with the same stacking orientation. Outside these crystalline regions, the surrounding areas were amorphous. Figure 3e shows the WSiN sublayer’s SAED pattern with diffuse halo rings indicative of a near-amorphous structure. Figure 3f depicts an XTEM image of the CrSiN/WSiN interface in sample 2L. A 20 nm-thick amorphous region is visible, implying that nucleation of CrN grains on the amorphous WSiN sublayer is difficult, and that the interface region between the two sublayers is either amorphous or composed of nanocrystalline domains below the resolution limit. 

**Figure 4:**
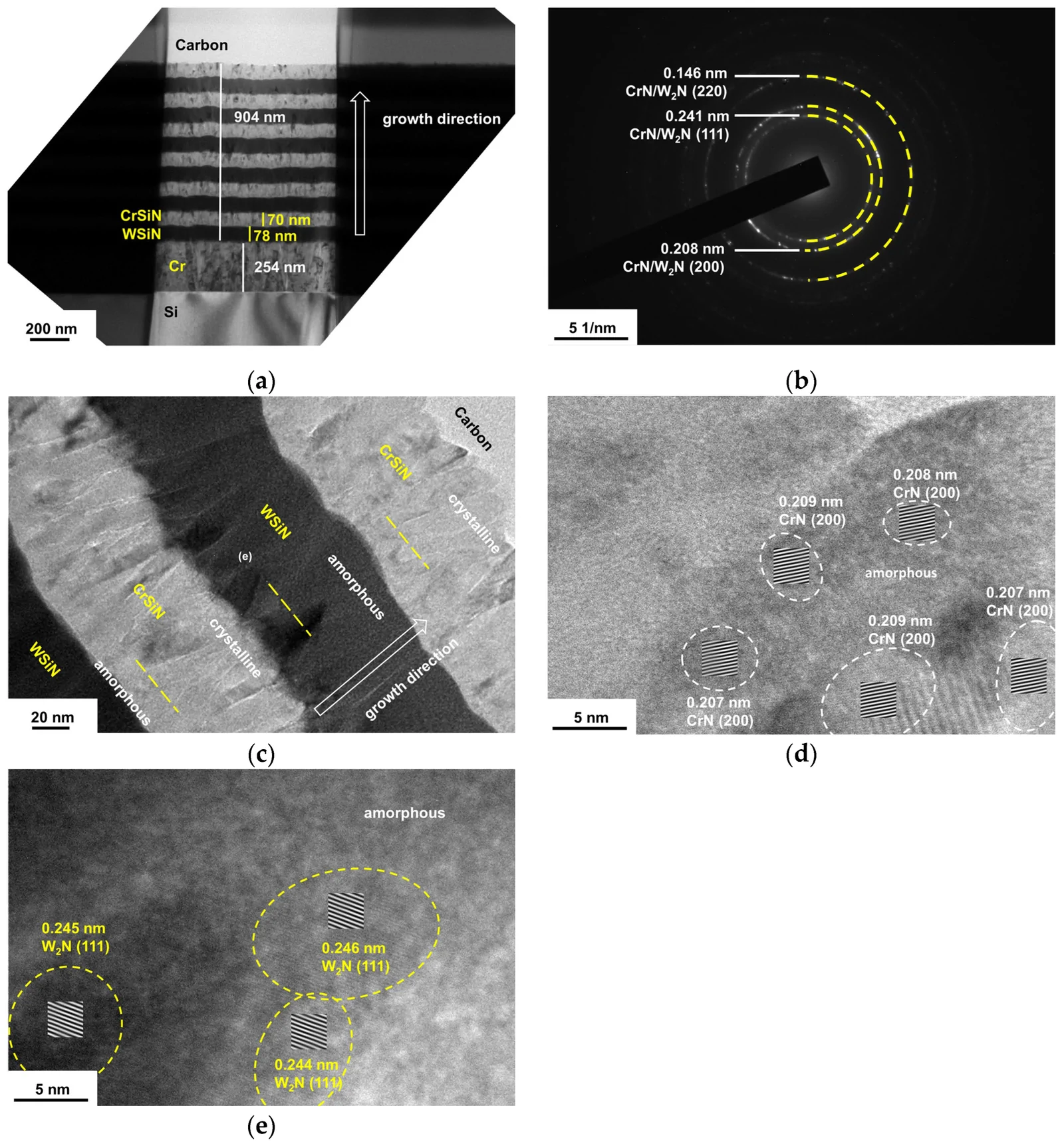
(**a**) The XTEM image presents the multilayer structure of sample 12L with alternately stacked WSiN and CrSiN sublayers. (**b**) The SAED pattern of sample 12L exhibits a crystalline FCC phase. (**c**) An XTEM image of the outermost four sublayers presents the crystalline and amorphous regions within them. (**d**) CrSiN and (**e**) WSiN sublayers’ HRTEM images exhibit crystalline regions at the nanoscale.

Figure 4a shows an XTEM image of sample 12L, consisting of alternating WSiN and CrSiN sublayers grown along the growth direction. The CrSiN sublayers appear brighter, an effect attributed to the lower atomic number and scattering factor of Cr compared to W. Well-defined interfaces between the WSiN and CrSiN sublayers are observed. The 12L sample comprises a 254-nm-thick Cr interlayer, six WSiN sublayers, and six CrSiN sublayers. Figure 4b presents the SAED pattern of sample 12L, revealing diffraction rings corresponding to the CrN and/or W_2_N phases, whose *d*-spacings are similar to those in the SAED pattern of sample 2L (Figure 3b). Figure 4c displays the outermost 4 stacking sublayers of sample 12L. The initial regions of CrSiN sublayers deposited on WSiN sublayers appear amorphous, identical to those reported for sample 2L (Figure 3f). The outer regions of the CrSiN sublayers are crystalline, with evident grain boundaries. In contrast, the initial regions of WSiN sublayers are crystalline as grown on CrSiN sublayers. The CrSiN sublayer’s HRTEM image shown in Figure 4d discloses nanocrystalline regions with crystallite sizes ranging from 4 to 8 nm, embedded within an amorphous matrix. The lattice fringes of 0.207–0.209 nm imply stacking of CrN (200) planes. Figure 4e displays an HRTEM image of the WSiN sublayer indicated in Figure 4c, and crystalline regions near the WSiN/CrSiN interface are observed, revealing lattice fringes with *d*-spacings of 0.244–0.246 nm, implying W_2_N (111) plane formation. The crystallinity of the stacked sublayer was affected by the sublayer beneath.

### 3.2. Mechanical Properties

**Figure 5:**
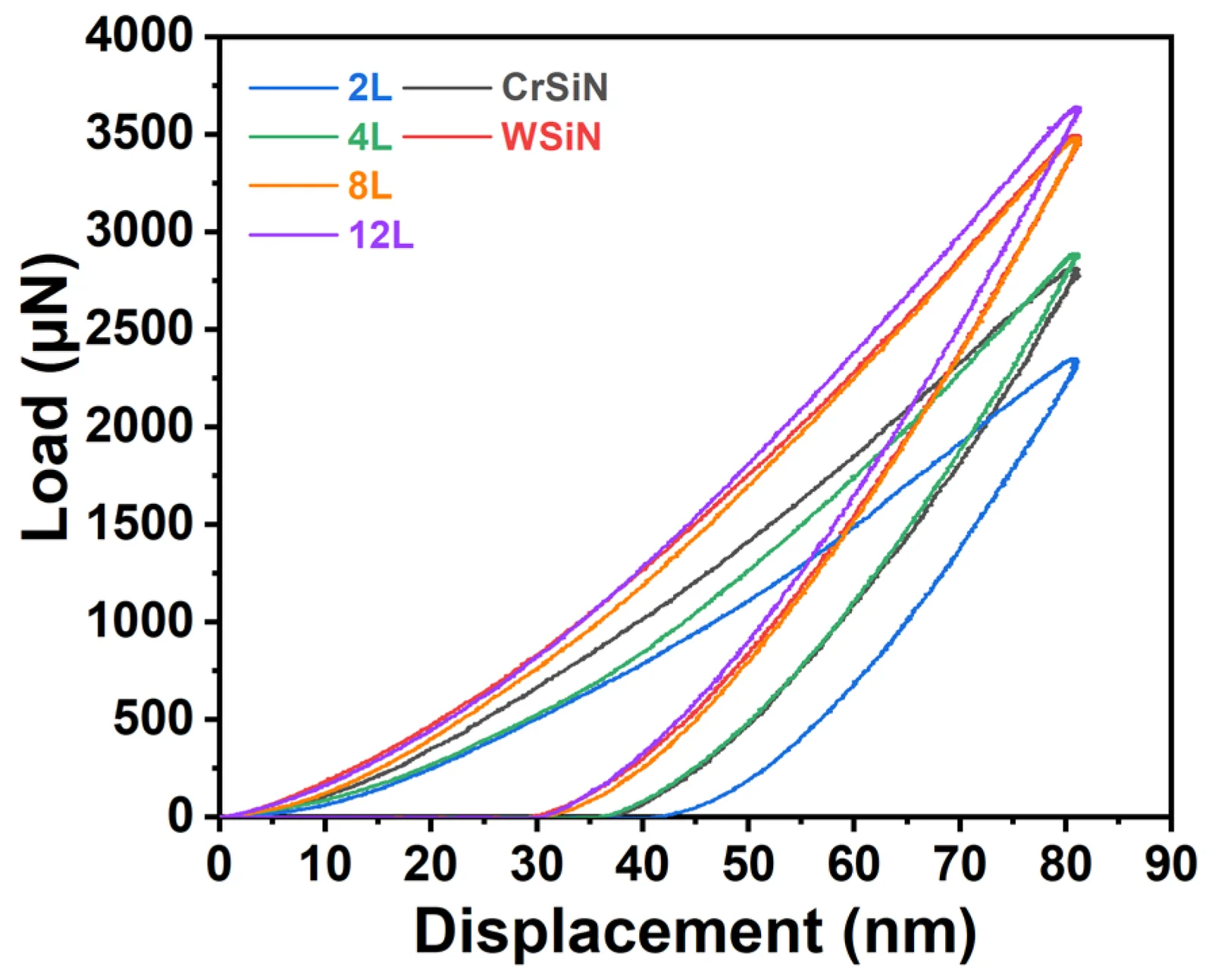
Load–displacement curves of the CrSiN/WSiN coatings with a maximal indentation depth of 80 nm.

Figure 5 shows the load–displacement curves of the indentation test with a maximal indentation depth of 80 nm. Table 2 records the mechanical properties of the monolithic films and multilayer coatings. The monolithic CrSiN film exhibits a hardness (*H*) of 14.5 GPa and elastic modulus (*E*) of 209 GPa. In contrast, the monolithic WSiN film shows a higher *H* of 19.7 GPa and *E* of 250 GPa. The hardness of sample 2L was 10.2 GPa, lower than the 14.5 GPa of the monolithic CrSiN film, which is attributed to the presence of an amorphous region during the initial deposition of the CrSiN sublayer. The *H* and *E* values of the CrSiN/WSiN multilayer coatings increase from 10.2 and 198 GPa to 15.1 and 220 GPa, 19.8 and 253 GPa, and 20.7 and 257 GPa, respectively, as the bilayer period decreases from 897 to 420, 205, and 143 nm. The hardness of sample 2L was dominated by the CrSiN sublayer when considering the indentation depth effect. In contrast, the WSiN sublayers evidently contributed to the coating’s hardness, with a small bilayer period. The influence of the soft and amorphous CrSiN part on the overall hardness of the multilayer coating was compensated by the harder and crystalline WSiN region, which resulted in the highest hardness of sample 12L compared to that of the monolithic and amorphous WSiN film. Correspondingly, the *H*/*E* ratio rises from 0.051 to 0.069, 0.078, and 0.081; the *H*^3^/*E*^2^ ratio increases from 0.027 to 0.072, 0.122, and 0.135; and the elastic recovery (W_e_) increases from 45% to 58%, 60%, and 61% with decreasing bilayer period. The enhancement in mechanical properties is attributed to the multilayer architecture, in which interfaces serve as effective barriers to dislocation motion, thereby increasing hardness through a strengthening effect [28,29,44]. Due to the mismatch in shear modulus between adjacent layers, dislocations are impeded at the interfaces, requiring higher stress to traverse the boundaries. Consequently, a higher interface density within the multilayer structure further suppresses dislocation glide across layers, improving mechanical performance. The CrSiN film exhibits a tensile residual stress of 0.15 GPa, whereas the WSiN film exhibits a compressive residual stress of −0.61 GPa, comparable to those reported in previous studies [14,15]. The CrSiN/WSiN multilayer coatings exhibit compressive residual stresses ranging from −0.14 to −0.38 GPa, indicating that the WSiN sublayers dominate the multilayer’s residual stress state.

**Figure 6:**
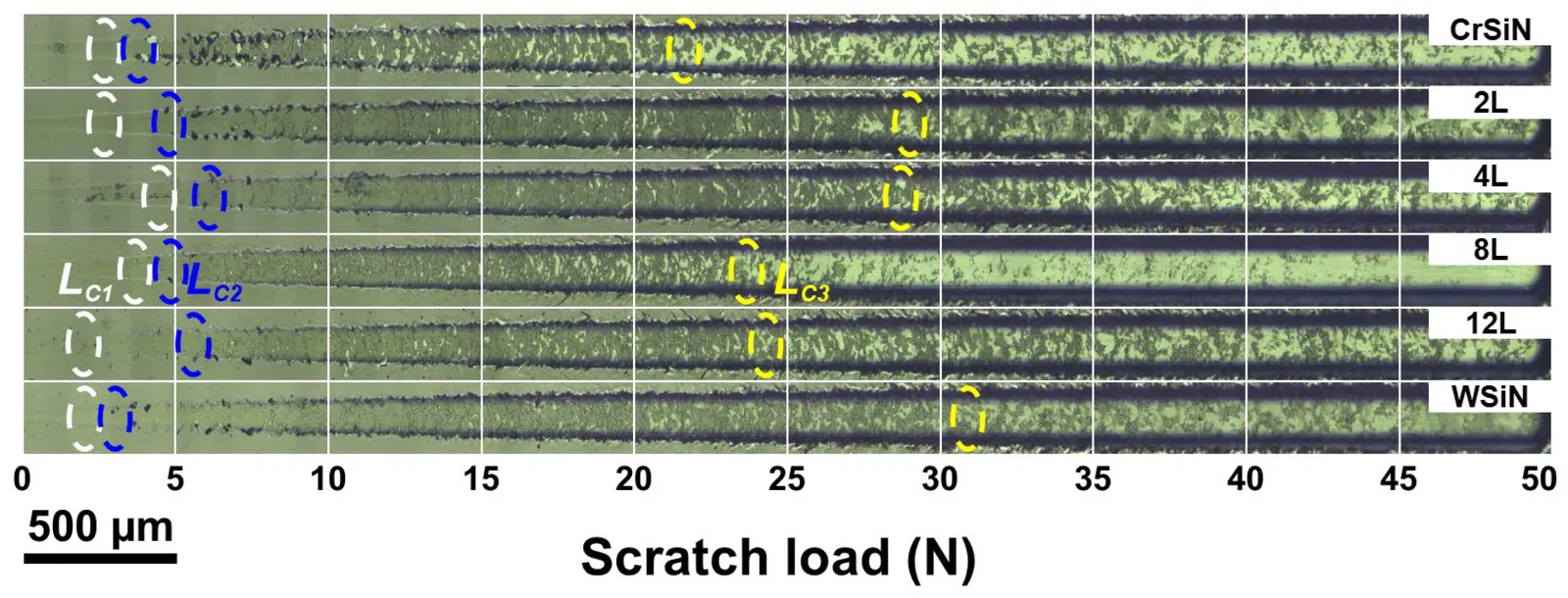
Scratch tracks of CrSiN/WSiN multilayer coatings prepared on SUS304 substrates after the scratch tests indicate the three critical loads *L_C1_*, *L_C2_*, and *L_C3_*.

Figure 6 shows the surface morphology of the scratch tracks observed using an optical microscope. Three critical loads (*L_C1_*, *L_C2_*, and *L_C3_*) [45] were labeled on the scratch tracks. *L_C1_* and *L_C2_* denoted the initial crack formation and adhesive failure, respectively. The critical load *L_C3_*, defined as the load at which the substrate becomes exposed, indicates the adhesion strength. The *L_C3_* values of samples 2L, 4L, 8L, and 12L were 29.5, 29.4, 24.2, and 24.7 N, respectively. The CrSiN/WSiN multilayer coatings exhibited an intermediate adhesion strength, falling between those of monolithic CrSiN (22.0 N) and WSiN (31.5 N) films. Among the multilayer samples, samples 2L and 4L exhibited better adhesion than samples 8L and 12L. The multilayer coatings with a lower stacking period exhibited inferior performance in the scratch test, which could be one of the reasons affecting their wear behavior.

Although the monolithic WSiN film demonstrated a higher hardness (19.7 GPa) than the CrSiN film (14.5 GPa), its wear resistance was inferior. After undergoing a 100 m wear distance, the wear rates of WSiN and CrSiN films were determined to be 4.16 × 10^−6^ and 8.46 × 10^−7^ mm^3^/Nm, respectively. All the CrSiN/WSiN multilayer coatings exhibited wear resistance comparable to or superior to that of the monolithic CrSiN film, as summarized in Table 3. Incorporating CrSiN sublayers significantly enhanced the multilayer’s wear resistance. All samples showed wear depths less than their coating thicknesses. Moreover, the wear depths were 25 and 95 nm for samples 2L and 4L, respectively, which were lower than the 451 and 188 nm observed for the outermost CrSiN sublayer (Figure 2). By contrast, the wear depth of sample 12L was 140 nm, which was comparable to its bilayer period of 143 nm. Notably, sample 2L demonstrated the highest wear resistance among all multilayer configurations. The low hardness and elastic modulus of sample 2L, ascribed to the amorphous region at the CrSiN/WSiN interface, make it more easily plastically deformed. In a previous study [46], the wear behavior of CrN/ZrB_2_ bilayer films has been evaluated. The structure of the soft CrN sublayer on the hard ZrB_2_ sublayer results in the bilayer films behaving like a soft film on a hard substrate [47]. The CrN phase, with high ductility [48], improves the wear resistance of CrN/ZrB_2_ bilayer films by plastic deformation [46]. The soft CrN sublayer protected the ZrB_2_ sublayer beneath it via shearing deformation. In this study, the CrSiN and WSiN sublayers in samples 2L and 4L, respectively, played roles similar to those of CrN and ZrB_2_ sublayers.

**Figure 7:**
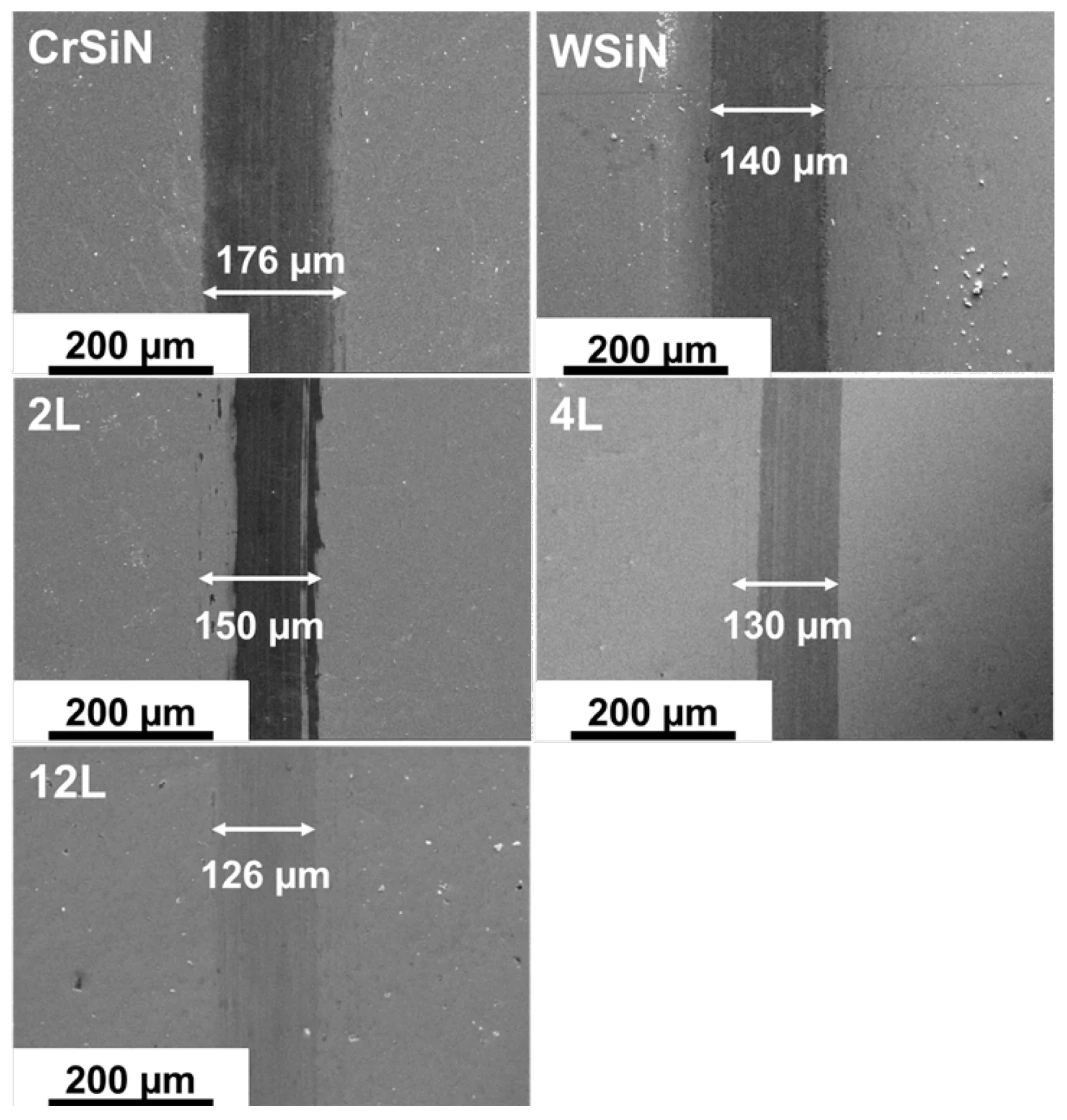
Surface images of the CrSiN/WSiN coatings after 100 m of sliding wear exhibit smooth wear tracks.

**Figure 8:**
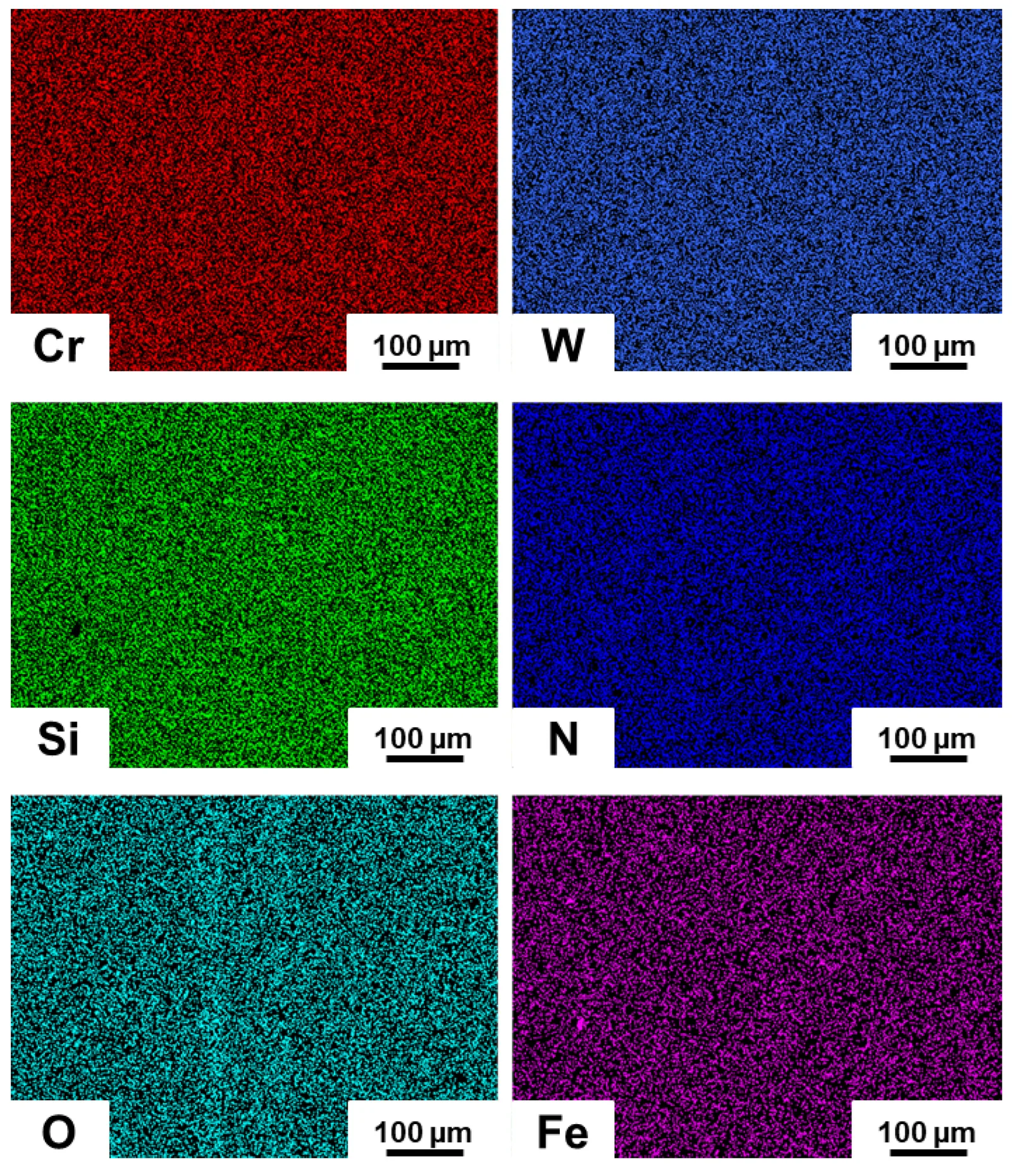
Elemental mappings of sample 12L without wear-through after the wear test exhibit homogeneous element distributions.

**Figure 9:**
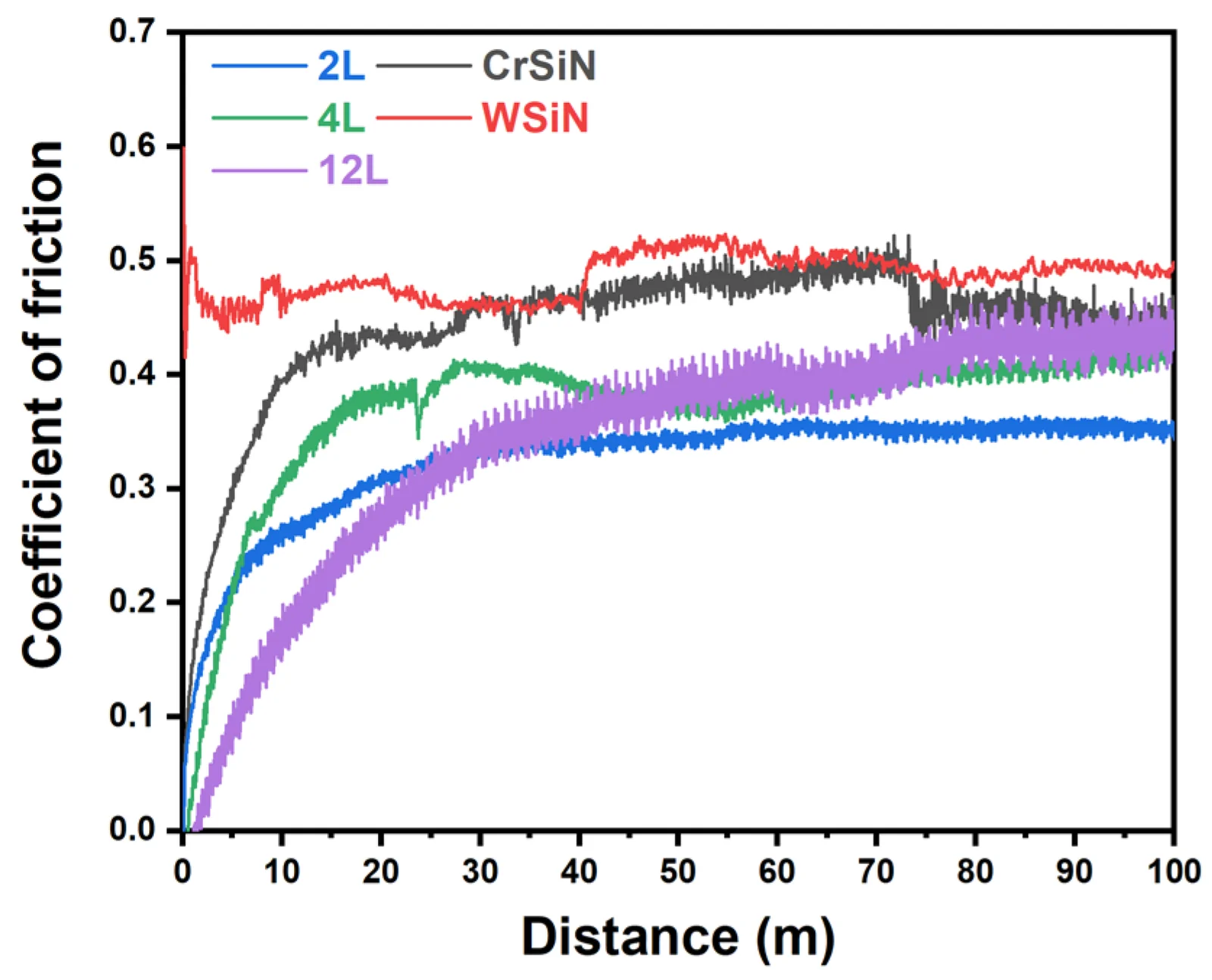
COF variations of the CrSiN/WSiN coatings during the wear tests.

Figure 7 illustrates the wear-track morphologies of the wear-tested monolithic films and multilayer coatings, as observed by SEM. All the tested samples revealed smooth wear tracks. Figure 8 presents the EDS analysis on sample 12L’s surface, also shown in Figure 7. The absence of Fe signals from the SUS304 substrate indicates that the coatings were not worn through. Locally, O aggregation was observed along the wear track. Among all multilayer coatings, sample 2L exhibited the best wear resistance, with a wear rate of 3.79 × 10^−7^ mm^3^/N·m. In contrast, a wear rate of 8.06 × 10^−7^ mm^3^/N·m for sample 12L was comparable to 8.46 × 10^−7^ mm^3^/N·m for the monolithic CrSiN film; however, sample 12L possessed the highest hardness of 20.7 GPa among the surveyed coatings, owing to the incorporation of WSiN sublayers in the multilayer coatings. The wear test results cannot be explained by conventional prediction rules based on *H*/*E* and *H*^3^/*E*^2^ indicators [49,50,51], which suggest that higher toughness and wear resistance are associated with higher *H*/*E* and *H*^3^/*E*^2^ values. Sample 12L exhibited higher *H*/*E* and *H*^3^/*E*^2^ values but lower wear resistance compared to sample 2L. A similar inconsistency was observed in a previous study evaluating the wear behavior of the (Zr_x_Ta_1−x_)B_y_/Cr/SUS304 construction [52]. The nanoindentation test examined film deformation at shallow depths. In contrast, the wear test evaluated the overall wear performance of the multilayer coatings, including their interlayer and adhesion to the substrate. Chen et al. [53] noted that the toughness of films with plasticity exhibited wear behavior that differed from that predicted by the *H*/*E* and *H*^3^/*E*^2^ indicators. Sample 2L exhibited a low *W_e_* of 45%, implying plastic deformation evidently affected the wear behavior. A similar low *W_e_* of 42% was observed for the CrSiN/WSiN bilayer film, with low *H*, *E*, *H*/*E*, and *H*^3^/*E*^2^ values, showing a wear depth less than the CrSiN sublayer thickness [36]. The coefficients of friction (COFs) of the CrSiN/WSiN multilayer coatings at a wear interval of 30–100 m, ranging from 0.35 to 0.40, were lower than those of the monolithic CrSiN (0.47) and WSiN (0.49) films. These COFs of the CrSiN/WSiN multilayer coatings varied smoothly (Figure 9).

## 4. Conclusions

In this study, CrSiN/WSiN multilayer coatings with various stacking periods were successfully fabricated and systematically evaluated regarding their structural characteristics, mechanical properties, and wear resistance. A decrease in the bilayer period enhances hardness, elastic modulus, and elastic recovery. Transmission electron microscopy revealed well-defined interfaces within the multilayer structure and confirmed the structural evolution between crystalline and amorphous regions during sublayer stacking. Sample 12L, with a stacking period of 143 nm, has the highest hardness and elastic modulus, at 20.7 and 257 GPa, respectively, accompanied by the highest elastic recovery, at 61%. Notably, sample 2L exhibits the best wear resistance. None of the multilayer coatings were worn through during the wear test and they exhibited low wear rates of 3.79–8.06 × 10^−7^ mm^3^/Nm. In summary, CrSiN/WSiN multilayer coatings with an optimized stacking period of 12 sublayers achieved higher hardness than monolithic CrSiN films and enhanced wear resistance comparable to that of monolithic WSiN films, making them promising candidates for advanced protective coating applications.

## Figures and Tables

**Table 1:** Thicknesses of the CrSiN/WSiN multilayer coatings.

Sample	*t_D_* ^a^ (min)		*T* ^b^ (nm)		*R_T_* ^c^
	CrSiN	WSiN	T_CrSiN_	T_WSiN_	T_CrSiN_/T_WSiN_
CrSiN	72	-	811	-	-
2L	36	42	451	446	1.01
4L	18	21	194 ± 8	226 ± 16	0.86
8L	9	10.5	93 ± 9	112 ± 3	0.83
12L	6	7	64 ± 3	79 ± 5	0.81
WSiN	-	84	-	957	-

^a^ *t_D_*: deposition time in each sublayer. ^b^ *T*: thickness. ^c^ *R_T_*: thickness ratio.

**Table 2:** Mechanical properties of the CrSiN/WSiN multilayer coatings.

Sample	*H* ^a^ (GPa)	*E* ^b^ (GPa)	*H*/*E*	*H*^3^/*E*^2^ (GPa)	*W_e_* ^c^ (%)	Residual Stress (GPa)
CrSiN	14.5 ± 0.6	209 ± 8	0.069	0.070	52	0.15 ± 0.08
2L	10.2 ± 0.8	198 ± 6	0.051	0.027	45	−0.24 ± 0.02
4L	15.1 ± 0.1	220 ± 2	0.069	0.072	58	−0.25 ± 0.01
8L	19.8 ± 0.8	253 ± 4	0.078	0.122	60	−0.38 ± 0.04
12L	20.7 ± 0.3	257 ± 3	0.081	0.135	61	−0.14 ± 0.01
WSiN	19.7 ± 0.2	250 ± 3	0.079	0.122	59	−0.61 ± 0.00

^a^ *H*: hardness. ^b^ *E*: elastic modulus. ^c^ *W_e_*: elastic recovery.

**Table 3:** Wear test results of the CrSiN/WSiN coatings.

Sample	Thickness (nm)	Wear Depth (nm)	Wear Width (um)	Wear Rate (mm^3^/Nm)	COF ^a^
CrSiN	1019	368	176	8.46 × 10^−7^	0.47
2L	1125	25	150	3.79 × 10^−7^	0.35
4L	1025	95	130	7.08 × 10^−7^	0.40
12L	1081	140	126	8.06 × 10^−7^	0.40
WSiN	1198	334	140	4.16 × 10^−6^	0.49

^a^ COF: average values of the coefficient of friction at wear distances of 30–100 m.

## Data Availability

The original contributions presented in this study are included in the article. Further inquiries can be directed to the corresponding author.
